# *Angiostrongylus* spp. in the Americas: geographical and chronological distribution of definitive hosts versus disease reports

**DOI:** 10.1590/0074-02760170226

**Published:** 2018-03

**Authors:** Romina Valente, Maria del Rosario Robles, Graciela T Navone, Julia I Diaz

**Affiliations:** 1 Instituto Nacional de Medicina Tropical, Puerto Iguazú, Misiones, Argentina; 2 Centro Científico Tecnológico La Plata (CONICET-UNLP), Facultad de Ciencias Naturales y Museo, Centro de Estudios Parasitológicos y de Vectores, Buenos Aires, Argentina

**Keywords:** American distribution, angiostrongyliasis, Angiostrongylus, disease reports

## Abstract

**BACKGROUND:**

Angiostrongyliasis is an infection caused by nematode worms of the genus *Angiostrongylus*. The adult worms inhabit the pulmonary arteries, heart, bronchioles of the lung, or mesenteric arteries of the caecum of definitive host. Of a total of 23 species of *Angiostrongylus* cited worldwide, only nine were registered in the American Continent. Two species, *A. cantonensis* and *A. costaricensis*, are considered zoonoses when the larvae accidentally parasitise man.

**OBJECTIVES:**

In the present study, geographical and chronological distribution of definitive hosts of *Angiostrongylus* in the Americas is analysed in order to observe their relationship with disease reports. Moreover, the role of different definitive hosts as sentinels and dispersers of infective stages is discussed.

**METHODS:**

The study area includes the Americas. First records of *Angiostrongylus* spp. in definitive or accidental hosts were compiled from the literature. Data were included in tables and figures and were matched to geographic information systems (GIS).

**FINDINGS:**

Most geographical records of *Angiostrongylus* spp. both for definitive and accidental hosts belong to tropical areas, mainly equatorial zone. In relation to those species of human health importance, as *A. cantonensis* and *A. costaricensis*, most disease cases indicate a coincidence between the finding of definitive host and disease record. However, in some geographic site there are gaps between report of definitive host and disease record. In many areas, human populations have invaded natural environments and their socioeconomic conditions do not allow adequate medical care.

**MAIN CONCLUSIONS:**

Consequently, many cases for angiostrongyliasis could have gone unreported or unrecognised throughout history and in the nowadays. Moreover, the population expansion and the climatic changes invite to make broader and more complete range of observation on the species that involve possible epidemiological risks. This paper integrates and shows the current distribution of *Angiostrongylus* species in America, being this information very relevant for establishing prevention, monitoring and contingency strategies in the region.

Angiostrongyliasis is an infection caused by nematode worms of the genus *Angiostrongylus* Kamensky 1905. The adult worms inhabit the pulmonary arteries, vena cava and right ventricle of the heart, bronchioles of the lung, or mesenteric arteries of the caecum of definitive host, which include rodents, tupaiids, mephitids, mustelids, procyonids, felids, or canids, and aberrantly in a range of avian, marsupial and eutherian hosts including humans (Anderson et al. 2010). Definitive hosts release first-stage larvae in the feces, which utilise slugs and/or aquatic or terrestrial snails as intermediate hosts. Gastropods are infected by ingestion or penetration of first-stage larvae; while definitive hosts are infected by ingestion of gastropods or their slime. Also, the transmission could involve ingestion of paratenic hosts (Cross 2004, Thiengo 2007, Spratt 2015).

Of a total of 23 species of *Angiostrongylus* cited worldwide, only nine were registered in the American Continent: *Angiostrongylus vasorum* (Bailliet, 1866), *Angiostrongylus cantonensis* (Chen 1935), *Angiostrongylus raillieti* Travassos 1927, *Angiostrongylus gubernaculatus* Dougherty 1946, *Angiostrongylus costaricensis* Morera and Céspedes 1971, *Angiostrongylus schmidti* Kinsella 1971, *Angiostrongylus morerai* Robles, Navone and Kinsella 2008, *Angiostrongylus lenzii* Souza, Simões, Thiengo et al. 2009 and *Angiostrongylus felineus* Vieira et al. 2013 (Kinsella 1971, Robles et al. 2008, Souza et al. 2009, Vieira et al. 2013, Spratt 2015).

Two species, *A. cantonensis* and *A. costaricensis*, causing neurological and abdominal angiostrongyliasis respectively, are considered zoonoses when the larvae accidentally parasitise man. There are many reports of these diseases in different American countries (Acha & Szyfres 2003, Maldonado Jr et al. 2010, Spratt 2015). *Angiostrongylus vasorum*, causing a respiratory pathology in wild and domestic canids, has veterinary importance (Koch & Willesen 2009). The rest of *Angiostrongylus* species only are known in wild animals and there are no data on their epidemiological potential (Spratt 2015, Robles et al. 2016).

Some studies (Kinsella 1971, Robles et al. 2016) suggest the low host specificity of *Angiostrongylus* spp. Besides that, other studies as Spratt (2015) warns that most of the reports of these species only reflect lack of opportunity or interest in examining nonurban and nonagricultural hosts.

It is puzzling that there has been no cases of eosinophilic meningoencephalitis or abdominal angiostrongyliasis in some points of the American distribution to date, even considering that the characteristics of the environment and the presence of several intermediate hosts (registered and potential) and wild definitive host allow the presence of different species of *Angiostrongylus* (Robles et al. 2008, 2016, Souza et al. 2009, Maldonado Jr et al. 2010).

As in most natural systems, the climate change affects physiology of hosts and parasite, altering survivorship, reproduction, and transmission, among other factors. In addition, in different parts of the world, the environment and socioeconomic systems are changing rapidly, modifying interactions among humans, animals, and their pathogens (Kutz et al. 2005, Salb et al. 2008).

In the present study, geographical and chronological distribution of definitive hosts of *Angiostrongylus* in the Americas is analysed in order to observe their relationship with disease reports. Moreover, the role of different definitive hosts as sentinels and dispersers of infective stages is discussed.

## MATERIALS AND METHODS

The study area includes the Americas (i.e. North, Central and South America, and Caribbean). First records of the parasite of *Angiostrongylus* spp. in definitive or accidental hosts were compiled from the literature (scientific literatures and book sections). When necessary, scientific names of mammal hosts have been updated following Wilson and Reeder (2005) and Weksler et al. (2006). Published disease reports in non-indexed journals or in internal articles of regional hospitals were reviewed and most of them included, however some cases were ignored for not showing clear evidence.

**Fig. 1:**
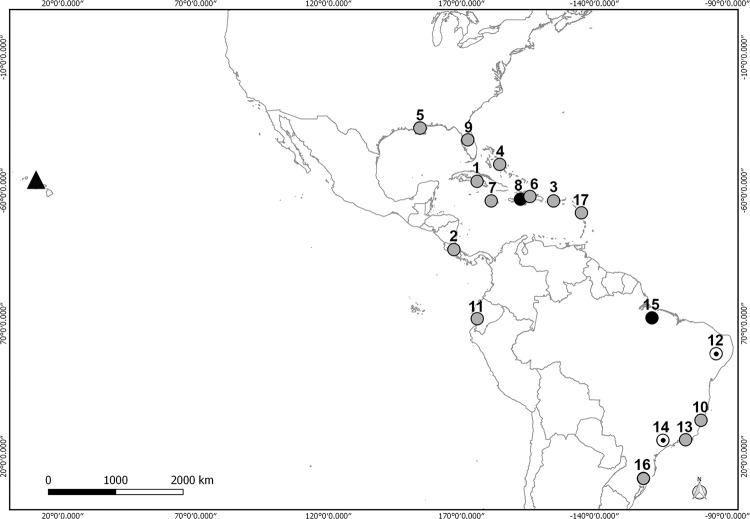
*Angiostrongylus cantonensis* in the Americas and Hawaii (▲) showing reports of adult parasites without disease (black points), and reports of adult parasite and disease (grey points) (references in Table I).

**Fig. 2:**
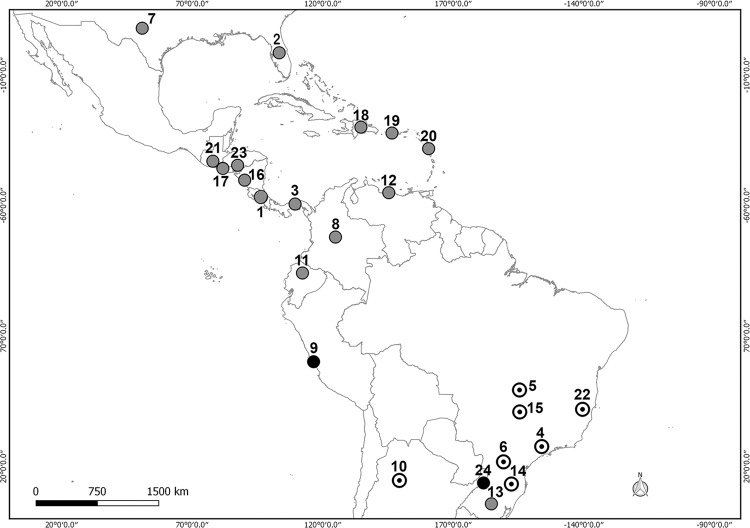
*Angiostrongylus costaricensis* in the Americas showing reports of adult parasites without disease (black points), report of disease (white point), and reports of adult parasite and disease (grey points) (references in Table II).

Data were included in Tables I–III and were matched to geographic information systems (GIS) using QUANTUM GIS (Version 2.10 PISA). Coordinates of geographical sites were obtained from the gazetteer GeoHack Web Application. The first records of adult parasite, disease, or both at once are showed in the maps with different symbols (Figs 1–2). Geographic sites that are located in the same state were plotted in a single point on the figures.

**TABLE I:** Reports of *Angiostrongylus cantonensis* in the Americas and in Hawaii, showing date of first report of adult parasite in chronological order, definitive hosts, and date of the first disease record in each geographical site. Numbers and symbol correspond to the references in Fig. 1

Nematode species	Reference in map	First record of adult parasite	Definitive host	First record disease	Accidental host	Geographical site	Literature consulted
*Angiostrongylus cantonensis*		1960	*Rattus rattus*	1960	*Homo sapiens*	Hawaii*	Alicata (1991)
			*Rattus norvegicus*				
	1	1977	*R. norvergicus*	1981	*H. sapiens*	Cuba*	Aguiar et al. (1981)
	2	1980	*Sigmodon hispidus*	1980	*H. sapiens*	Costa Rica*	Núñez and Mirambell (1981)
			*R. rattus*				
	3	1984	*R. rattus R. norvegicus*	1986	*H. sapiens*	Puerto Rico*	Anderson et al. (1986)
	4	1987	*R. rattus*	1990	*Hylobates lyr*	Bahamas, USA	Vargas et al. (1992)
			*R. norvegicus*				
	5	1988	*R. norvergicus*	1992	*Alouatta caraya*	Louisiana, USA	Campbell and Little (1988), Cross (2004)
			*Podomys floridanus^a^*		*Lemur variegatus*		
			*Didelphis virginiana*		*Cercophitecus*		
			*R. norvergicus*		*talapon*		
			*Varecia variegata*		*H. sapiens*		
	6	1992	*R. rattus*	1992	*H. sapiens*	Republica	Vargas et al. (1992)
						Dominicana*	
	7	1994	*R. rattus*	1994	*H. sapiens*	Jamaica*	Lindo et al. (2002)
			*R. norvegicus*				
	8	2002	*R. rattus*	-	*-*	Puerto Principe, Haiti	Raccurt et al. (2003)
			*R. norvergicus*				
	9	2003	*R. rattus*	2003	*H.lyr*	Florida, USA	Cross (2004), Duffy et al. (2004)
			*R. norvergicus*				
	10	2007	*R. norvegicus*	2007	*H. sapiens*	Espírito Santo, Brazil	Caldeira et al. (2007)
	11	2008	*R. norvergicus*	2008	*H. sapiens*	Guayas, Ecuador	Martini-Robles and Dorta Contreras (2016)
							
	12	-	*-*	2009	*H. sapiens*	Pernambuco, Brazil	Lima et al. (2009)
	13	2010	*R. norvergicus*	2007	*H. sapiens*	Rio de Janeiro, Brazil	Simões et al. (2011)
							Morassutti et al. (2014)
	14		*-*	2010	*H. sapiens*	São Paulo, Brazil	Espírito Santo et al. (2013)
	15	2013	*R. rattus*	-	*-*	Pará, Brazil	Morassutti et al. (2014)
			*R. norvergicus*				
	16	2013	*R. norvergicus*	2013	*H. sapiens*	Rio Grande do Sul,	Cognato et al. (2013)
						Brazil	Morassutti et al. (2014)
	17	2013	*R. rattus*	2013	*H. sapiens*	Guadalupe	Dard et al. (2017)
			*R. norvegicus*				

* the country capital coordinates were used since the localities were not provided. Registered as *a: Neotoma floridanus.*

**TABLE II:** Reports of *Angiostrongylus* in the Americas, showing date of first report of adult parasite in chronological order, definitive hosts, and date of the first disease record in each geographical site. Numbers correspond to the references in Fig. 2

Nematode species	Reference in map	First record of adult parasite	Definitive host	First record disease	Accidental host	Geographical site	Literature consulted
*Angiostrongylus costaricensis*	1	1971	*Rattus norvergicus*	1971	*Homo sapiens*	Costa Rica*	Morera and Cespedes (1971)
			*Sigmodon hispidus*				Monge et al. (1978)
			*Rattus rattus*				Alfaro-Alarcon et al. (2015)
			*Nasua narica*				
			*Canis familiaris*				
	2	1971	*S. hispidus*	1971	*Aotus*	Florida, USA	Ubelaker and Hall (1979)
			*Didelphis virginiana*		*nancymaae*		
			*Procyon lotor*				
			*Symphalangus*				
			*syndactylus^b^*				
	3	1973	*S. hispidus*	1973	*H. sapiens*	Panama*	Kaminsky (1996)
			*R. rattus*				
			*Liomys adspersus*				
			*Oligoryzomys*				
			*fulvescens^c^*				
			*Zygodontomys*				
			*brevicauda^d^*				
	4		-	1975	*H. sapiens*	São Paulo, Brazil	Zilliota Jr et al. (1975)
	5		-	1980	*H. sapiens*	Brasilia, Brazil	Agostini et al. (1984)
	6		-	1982	*H. sapiens*	Paraná, Brazil	Ayala (1987)
	7	1979	*S. hispidus*	1994	*H. sapiens*	Texas, USA	Miller et al. (2006)
	8	1981	*S. hispidus*	1981	*H. sapiens*	Colombia*	Malek (1981)
			*Melanomys*				
			*caliginosus^e^*				
	9	1982	*Saguinus mystax*	1982	-	Peru*	Sly et al. (1982)
	10		-	1982	*H. sapiens*	Tucumán, Argentina	Demo and Pessat (1986)
	11	1983	*R. rattus*	1983	*H. sapiens*	Ecuador*	Kaminsky (1996)
			*R. norvergicus*				
	12	1985	*Proechimys* sp.	1985	*H. sapiens*	Venezuela*	Kaminsky (1996)
	13	1990	*0. nigripes*	1983	*H. sapiens*	Rio Grande do Sul,	Agostini et al. (1984)
			*Sooretamys angouya^f^*			Brazil	Graeff-Teixeira et al. (1990)
	14		-	1987	*H. sapiens*	Santa Catarina, Brazil	Ayala (1987)
	15		-	1991	*H. sapiens*	Minas Gerais, Brazil	Rocha et al. (1991)
*Angiostrongylus costaricensis*	16	1991	*S. hispidus*	1991	*H. sapiens*	Nicaragua*	Duarte et al. (1991)
	17	1992	*S. hispidus*	1992	*H. sapiens*	El Salvador*	Kaminsky (1996)
	18	1992	*R. rattus*	1992	*H. sapiens*	Republica Dominicana*	Maldonado Jr et al. (2012)
			*R. norvergicus*				
	19	1992	*R. rattus*	1992	*H. sapiens*	Puerto Rico*	Maldonado Jr et al. (2012)
			*R. norvegicus*				
	20	1992	*R. rattus*	1992	*H. sapiens*	Guadalupe*	Kaminsky (1996)
			*R. norvergicus*				
	21	1994	*S. hispidus*	1994	*H. sapiens*	Guatemala*	Kaminsky (1996)
	22		*-*	1995	*H. sapiens*	Espírito Santo, Brazil	Pena et al. (1995)
	23	1996	*S. hispidus Peromyscus* spp.	1972	*H. sapiens*	Valle de Yegüare, Honduras	Zuñiga et al. (1983) Kaminsky (1996)
			*Mus musculus*				
	24	2008	*Akodon montensis*	-	*-*	Misiones, Argentina	Robles et al. (2016)

* the exact locality were not provided. Registered as *b: Hylobates syndactylus; c: Oryzomys fulvescens; d. Zygodontomys microtinus; e: Oryzomys caliginosus; f. Oryzomus ratticeps.*

**Fig. 3:**
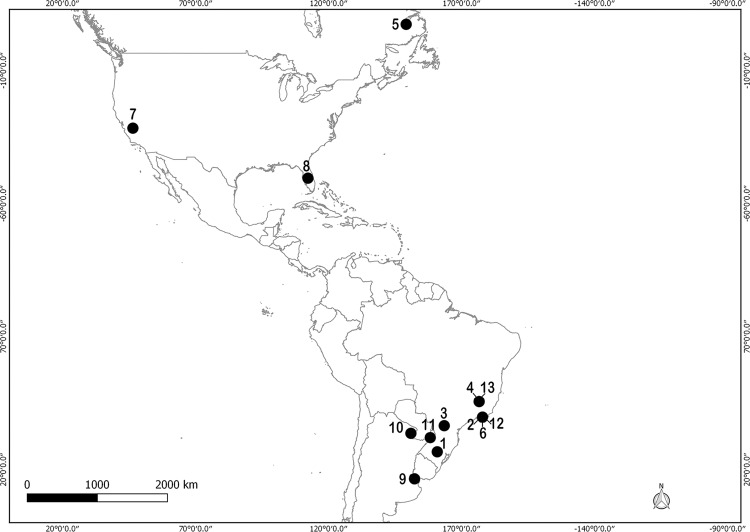
*Angiostrongylus* spp. (except *A. cantonensis* and *A. costaricensis*) in the Americas (references in Table III).

**TABLE III:** Reports of *Angiostrongylus* spp. (except *A. cantonensis* and *A. costaricensis)* in the Americas, showing date of first report of adult parasite in chronological order, definitive hosts, and date of the first disease record in each geographical site. Numbers correspond to references in Fig. 3

Nematode species	Reference in map	First record of adult parasite	Definitive host	First record disease	Accidental host	Geographical site	Literature consulted
*Angiostrongylus vasorum*	1	1961	*Canis familiaris*			Rio Grande do Sul, Brazil	Duarte et al. (2007)
	2	1962	*C. familiaris*			Rio de Janeiro, Brazil	Duarte et al. (2007)
			*Cerdocyon thous*				
	3	1985	*C. familiaris*			Paraná, Brazil	Duarte et al. (2007)
	4	1985	*C. familiaris*			Minas Gerais, Brazil	Duarte et al. (2007)
			*Dusicyon vetulus*				
	5	2000	*Vulpes vulpes*			Newfoundland, Canadá	Jeffery et al. (2004)
			*C. familiaris*				
*Angiostrongylus raillieti*	6	1927	*Cerdocyon thous azarae*			Rio de Janeiro, Brazil	Vieira et al. (2013)
			*C. familiaris*				
			*Nasua nasua*				
*Angiostrongylus gubernaculatus*	7	1971	*Taxidea taxus*			California, USA	Faulkner et al. (2001)
			*Urocyon littoralis*				
*Angiostrongylus schmidti*	8	1971	*Oryzomys palustris*			Florida, USA	Kinsella (1971)
*Angiostrongylus morerai*	9	2008	*Akodon azarae*			Buenos Aires, Argentina	Robles et al. (2008) Robles et al. (2016)
			*Akodon dolores*				
			*Deltamys kempi*				
	10	2016	*Necromys lasiurus liciae*			Formosa, Argentina	Robles et al. (2016)
			*Calomys callosus*				
			*Akodon azarae bibianae*				
	11	2016	*Akodon montensis Sooretamys angouya*			Misiones, Argentina	Robles et al. (2016)
*Angiostrongylus lenzii*	12	2009	*Akodon montensis*			Rio de Janeiro, Brazil	Souza et al. (2009)
*Angiostrongylus felineus*	13	2013	*Puma yagouaroundi*			Minas Gerais, Brazil	Vieira et al. (2013)

## RESULTS

In 36 geographic sites, at least one of the nine species of *Angiostrongylus* was reported. While disease records by angiostrongyliasis were obtained in 28 geographic sites. The Pan-American distributions of *Angiostrongylus* were five species in North America, two in Central America, seven in South America, and two in Caribbean (Tables I–III, Figs 1–3).

*A. cantonensis* was recorded in 16 geographic sites along the continent, six definitive host species and five accidental species affected by disease (Table I, Fig. 1).

On the map the distribution of this species is concentrated mostly in the area between the 29°58′0″N, 90°3′0″W and 27°16′12″S, 50°29′24″W coordinates. Seven points are located in South America, of which six are in Brazil and one in Ecuador (Fig. 1). Of the total records, only four do not indicate a coincidence between the finding of definitive host and disease record, of which three are in Brazil and one in Haiti (Table I).

In the case of *A. costaricensis*, the parasite was recorded in 24 geographic sites, 19 definitive hosts and only two accidental species affected by angiostrongyliasis (Table II, Fig. 2). In the map the distribution of this species is concentrated mostly in the area between the 31°0′0″N, 100°0′0″W and 29°45′36″S, 40°28′48″W coordinates. Thirteen points are located in South America, of which seven are in Brazil, two in Argentina and one each in Peru, Ecuador, Colombia and Venezuela (Fig. 2). The only record of definitive host in Argentina corresponds to one finding in Misiones province. This report is located 1300 km from the case disease in Tucumán province, and 500 km away from diseases cases in Brazil (Fig. 2).

In relation with those species of importance for the human health, most disease cases indicate a coincidence with the finding of definitive host. However, the period between the finding of definitive hosts and disease cases was 2-4 years for *A. cantonensis* and 7-24 for *A. costaricensis*.

The remaining seven *Angiostrongylus* species were recorded parasitising members of Marsupialia, Rodentia and Carnivora, including a total of 13 geographic sites (Table III, Fig. 3). *A. vasorum* has veterinary importance and was registered in four definitive hosts species and five geographic sites. This last species was recorded in a single locality from Canada and four from Brazil. Among those *Angiostrongylus* species parasitising wild carnivores, *A. raillieti* and *A. felineus* were recorded in Brazil, while *A. gubernaculatus* in USA. Among wild rodent parasitic species, *A. schmidti* was recorded in USA, while *A. lenzii* was recorded in Brazil and *A. morerai* in Argentina (Table III, Fig. 3).

## DISCUSSION

The present study has considered the first record of the adult nematode and the first record of disease as a way of evaluating the chronological distances between them. Additionally, the results in figures and tables showed both geographical distribution and host range of each *Angiostrongylus* species.

In 1960, two cases of eosinophilic meningitis caused by *A. cantonensis* were recorded in Hawaii. Notably, the definitive hosts were recorded almost at the same time as the disease. Although, this site is located in a different biogeographic region from those of the American continent (Neotropic and Neartic), the island belongs politically to USA. The constant flow of boats and people between the island and continent could have benefited the dispersion of the parasite (Cowie 2013).

A case of disease by enteric and lymphatic granulomas caused for Strongylida parasite was observed in Costa Rica in 1952 (Céspedes et al. 1967, Morera 1967). Later, the same authors, observed other similar clinical cases and the etiological agent, and described the species as *A. costaricensis*, considering the man as an accidental host without mentioning the possible definitive hosts (Morera & Céspedes 1971). Since 1972 different definitive hosts of this parasite were recorded, counting a total 19 hosts species (Kaminsky 1996, Romero-Alegría et al. 2014).

In many areas, mainly tropical, human populations have invaded natural environments and their socioeconomic conditions do not allow adequate medical care. Many cases could have gone unreported or unrecognised throughout history (Spratt 2015). Moreover, the population expansion and the climatic changes, invite to make broader and more complete range of observation on the species that involve possible epidemiological risks.

The Pan-American distribution of *Angiostrongylus* includes nine species. To date, a total of 33 definitive host species, seven accidental host species, and more than 20 intermediate host species have been recorded for those species of human health importance (*A. cantonensis*, *A. costaricensis*) (Grewal et al. 2003). For the remaining *Angiostrongylus* species, several definitive hosts and very few intermediate hosts have been registered. So, the advance in the study of intermediate hosts will be in relation to the knowledge of the definitive hosts and vice versa.

People usually become infected by eating raw or undercooked food contaminated with the larvae of *A. cantonensis* and *A. costaricensis*, or when they manipulate intermediate hosts for fishing (Ping-Wang et al. 2008, Romero-Alegría et al. 2014). In particular, a greater number of cases are expected in countries where intermediate hosts come into frequent contact with humans. It can be observed in the maps that *A. cantonensis* presents a distribution related with areas in which this feeding habit is present (e.g. Ecuador, Jamaica), being the main intermediate hosts reported *Lissachatina fulica*, *Subulina octona*, *Bradybaena similaris*, *Pomacea* sp., among others (Grewal et al. 2003, Thiengo 2007). In the case of *A. costaricensis*, although the geographical distribution is similar to *A. cantonensis*, the geographic and host records include a broader range. This interesting observation give rise to different hypotheses: (1) greater susceptibility of definitive hosts, increasing the probability of dispersing this species; (2) the site of infection of the adult parasite benefits its finding (gastrointestinal tracts of rodents are more studied than lung and heart MRR pers. obs.), so the distribution of *A. cantonensis* could be underestimated with respect to *A. costaricensis*. Future models of distribution, as well as experimental studies, would be helpful to clarify the epidemiological risks of contact with the intermediate hosts. Meanwhile, the present work advances and discusses the role of the definitive hosts as dispersers of *Angiostrongylus* species, taking account their role as sentinels of angiostrongyliasis.

Robles et al. (2016) and Spratt (2015) suggest that the diversity of *Angiostrongylus* species, as well the range of hosts, is underestimated. This situation could be due to the lack of interest in studying wild species and/or inadequate instruction for the detection of *Angiostrongylus*. These observations, added to the low specificity recorded for many species of this genus (Kinsella 1971, Robles et al. 2016), generate questions in relation to human health risks that involve some species that have not reported disease yet (e.g. *A. morerai*, *A. schmidti*, *A. lenzii*). The precarious state of knowledge about these species in America can be observed in the tables and on the map provided in this update.

In this work, the relationship between the definitive host records and the registered cases by angiostrongyliasis was observed. In this sense, in the bulk of cases the reports of diseases show correspondence with the findings of the adult parasite in the definitive host.

In Argentina, *A. costaricensis* was recorded only in one rodent species (*A. montensis*) in Misiones province (Robles et al. 2016), while a unique case of disease was reported in Tucumán province (Demo & Pessat 1986). The characteristics of the environments and climatic conditions of both geographic sites are very different, and the chronological distance between these reports is 30 years. This time exceeds the periods observed in the rest of the records for this species (0-24 years). The lack of reports on accidental and definitive hosts in Argentina is paradoxical, especially considering that there is not accurate data on the provenance of the patient in the only human case recorded. However, several human cases registered in Brazil since 1990 are located 500 km from Misiones province. Thus, the potential risk increases considering that the boundaries between human and wild animal's populations in the Atlantic forest are becoming increasingly diffuse. In addition, the main intermediate hosts registered for this two *Angiostrongylus* species (i.e. *L. fulica* and *Phyllocaulis variegatus*) are present in Argentina. However, no *Angiostrongylus* larvae were found in these mollusks to date (Valente et al. 2017).

This paper integrates and shows the current distribution of *Angiostrongylus* species in the Americas, being this information very relevant for establish prevention, monitoring and contingency strategies in the region.
